# The Pathologist Job

**DOI:** 10.1007/s13187-024-02492-z

**Published:** 2024-08-27

**Authors:** Konstantin Bräutigam

**Affiliations:** Institute of Tissue Medicine and Pathology, University of Bern, Murtenstrasse 31, 3008 Bern, Switzerland

**Keywords:** Pathology, Education, Medical training, Perspective

“Don’t you see patients?” “Are you even a doctor?” Many people, both those without medical training and medical professionals, do not seem to have a comprehensive understanding of the field of pathology [1, 2]. Pathology is a medical specialty that has long been overlooked or underappreciated [3] and is desperately seeking new talent [4]. The term “pathology” is derived from the Greek words “πάθος” (*pathos*) and “-λόγος “/”-λογία” (-*logos/-logia*), literally translated as “words for the suffering” or “study of disease,” and this is what fascinated me the most before, during, and after medical training. What are the mechanisms of disease, which pathways are involved, and how do they all fit together and make sense?

Over the past few centuries, the field of pathology has been inextricably linked with the visual imagery of dissection and the practice of clinical autopsy. This macabre perception of a pathologist’s job has now partly changed. However, manual dissection (commonly referred to as “macroscopy” or “macroscopic tissue examination”)—such as the embedding and orientation of tissue biopsies or the cutting of surgical specimens—still plays a central role. The dissector is by nature curious in his role to determine the ultimate cause of death, to discover unknown diseases, sometimes only at the microscopic level. Disease can inspire awe. The investigator can inspire reverence for life and the patient.

During my first few weeks as an intern, I found myself peering through the eyepieces like a detective. The attending handed me a stack of cases, mostly gastrointestinal biopsies. “Here, young man, get on with these, I will be with you in a few hours,” and off he went. I sat there, behind my microscope, looking at the slide. I felt uneasy. Even though I was just looking at a piece of glass, I was shaking with respect. The clinical information was sparse: “Biopsy—sigmoid colon—suspected malignancy.” Despite my fierce determination, I could not solve the case and give adequate answers to the questions posed. I could have interpreted a chest X-ray, an electrocardiogram, a blood gas analysis, but not this tiny piece of tissue. I was too inexperienced and could not even make sense of the structures I saw. Late at night, in the sweat of my brow, I thought about all the diverse things I had learned in medical school. But my apparent struggle to find the exact right answers made me feel useless and defeated.

In the dissecting room, I was intrigued by the variety and aesthetic dimension of each surgical specimen. Surgical pathology—the inspection, description, and processing of surgical specimens of any kind (neoplastic and not), for example—is a discipline that is not taught at university. Unfortunately, in the beginning, everything that could go wrong went wrong. I had no idea about specimen examination, adequate tissue sampling, and surgical margins. “Could this be a margin? Maybe I should ink that area,” with the principles of surgery in mind. Resection margins are of exceptional importance [5]. But what is a margin? The shaggy formalin-fixed parametria next to the uterine body, the vascular groove running next to the pancreatic neck, the tiny luminal structure on the femoral head? I struggled to concentrate. Nevertheless, I kept in mind the beauty of each specimen and its owner: my patient.

Pathology is a diverse and vast field. From cytology, i.e., the diagnostic assessment of cells in smears and body fluids, to histological examination to intraoperative (often) diagnostic frozen sections to molecular pathology, all share the need for investigative curiosity. Pathologists are crucial to a patient-centered healthcare approach, exemplified by their importance in interdisciplinary tumor boards. While histology (mainly hematoxylin-and-eosin-stained slides) serves as the mainstay of diagnostic routine and allows for adequate architectural and cytological assessment of tissue, molecular pathology is revolutionizing the diagnostic process and the classification of disease. Major advances in sequencing, epigenetics, and computational analysis led to the discovery, re-classification, and improved characterization of disease. Artificial intelligence and machine learning are paving the way for pathology to finally go (more) digital. Finally, tissue multiplexing, i.e., the synchronous detection and spatial visualization of multiple regions of interests, will revolutionize the future of the field.

But pathology is not only a diagnostic academic discipline, it is also an art. In histopathology, pattern recognition is fundamental, especially when examining histological slides. Tissue patterns can be both cellular and architectural. Spatial perception and thinking in terms of criteria-based categories are crucial for effective interpretation of histological samples. Some morphological phenomena are repetitive (e.g., the typical histological phenotype of a well-differentiated squamous cell carcinoma), but there are surprises such as disease variants or dedifferentiation. Detail and subtlety are essential (6).

When I signed out cases with my superiors, I understood the roughness of the profession. We were chasing a diagnosis and trying to answer the clinicians’ questions. What is malignant? What is reactive? The interobserver variability, i.e., the level of (dis)agreement between different examiners, could be enormous. What are the implications of my report? Is it dysplasia? Is it invasive growth? Is it a positive resection margin? Am I just being sloppy in detecting what I should have? Things could get complex, and I knew I would be giving reports with far-reaching consequences. But I had to make decisions.

Macro- and microscopic examination is different from clinical examination. Tissue interpretation often lacks the context of the whole patient. Finding the single cancer cell among thousands of non-neoplastic cells can be detective work with a challenging level of meticulousness. In our daily work, we are confronted with strict tissue processing and reporting protocols, especially in the case of malignant diseases, and a huge caseload. A diagnosis can be subject to debate and interpretation. But there is no room for wiping someone else’s hands clean. Resection margin accuracy and diagnostic discrepancies must be identified and reported, as must misdiagnoses. Slides must be reviewed responsibly. The opposite can be detrimental, because a patient’s fate is at stake. A diagnosis, whether malignant or not, can be a stigma and potentially a decision with major consequences. A diagnosis can determine therapy. Therapy can mean aggressive chemotherapy, radiation or debilitating surgery.

After the first few months of pathology residency, I felt exhausted. Despite the steep learning curve and my individual professional growth, I could not appreciate the immediate impact and consequences of my work. Were my diagnoses correct or was I robbing my patient of months? The patient was invisible. Sometimes, this was a good thing. Seeing patients can trigger a diagnostic suspicion bias. Empathy can interfere with objectivity. This is part of the harsh and cruel environment, but also part of the precise and meticulous nature of an excellent pathologist. As such, pathology deserves its rightful place in the broad spectrum of diagnostic disciplines, especially given its critical role in precision medicine and targeted therapy.
